# P034 Refractory giant cell arteritis: challenges of remission maintenance in the setting of multiple DMARD intolerance

**DOI:** 10.1093/rap/rkag113.064

**Published:** 2026-11-03

**Authors:** Arjun Narayana, Kirishananth Rajaseelan, Anupama Nandagudi

**Affiliations:** Mid and South Essex NHS Foundation Trust, Basildon, United Kingdom; Mid and South Essex NHS Foundation Trust, Basildon, United Kingdom; Mid and South Essex NHS Foundation Trust, Basildon, United Kingdom

## Abstract

**Introduction:**

Giant cell arteritis (GCA) is a serious inflammatory vasculitis often arising in patients with polymyalgia rheumatica (PMR), presenting with profound morning stiffness and girdle pain. Since untreated GCA can rapidly lead to arteritic anterior ischaemic optic neuropathy and irreversible visual loss, high-dose glucocorticoids remain the cornerstone of initial therapy. However, long-term steroid exposure carries substantial morbidity, making steroid-sparing DMARD therapy essential for sustained remission. This case report illustrates the complex, decade-long challenge of managing PMR evolving into GCA in a patient experiencing intolerance to every DMARD attempted, ultimately preventing successful steroid withdrawal and complicating disease control.

**Case description:**

Our patient, a woman in her 50s, initially presented with bilateral shoulder and pelvic girdle pain consistent with PMR. Her symptoms responded well to treatment, and remission was maintained until recurrence seven years later. In her early 60s, she developed severe, relapsing GCA characterised by intense temporal pain and jaw claudication, leading to her death in her late 60s. Ultrasound confirmed inflammatory wall thickening of the temporal artery in both transverse and longitudinal sections.

Management was complicated by chronically elevated CRP and ESR, making biochemical confirmation of flares less reliable. She frequently relapsed during steroid tapering, necessitating prolonged courses of prednisolone—often exceeding one year—and intermittent methylprednisolone injections. Long-term glucocorticoid exposure resulted in significant morbidity, including hypertension, obesity, poorly controlled diabetes, depression, anxiety, and suspected adrenal insufficiency.

Multiple DMARDs were trialled with limited success due to intolerance or infection. Sulphasalazine caused dose-dependent rash, and hydroxychloroquine was discontinued due to visual deterioration. Methotrexate triggered shingles, mucositis, diarrhoea, and exertional dyspnoea upon both initial use and re-challenge. Tocilizumab was considered but avoided since her recurrent diverticulitis posed an unacceptable risk of bowel perforation. Leflunomide and sulphasalazine were repeatedly interrupted due to infections. Azathioprine, initiated after ENRAD consultation, was similarly limited by severe diverticulitis and recurrent infections.

She was later referred to the BIOVAS trial team for non-ANCA vasculitis. Biologic options including adalimumab and abatacept were discussed; adalimumab was initiated, while abatacept was unavailable due to supply shortages. Despite expert recommendations to consider abatacept or baricitinib, she remained on adalimumab until her passing from small-bowel ischaemia a decade after her initial PMR diagnosis.

This case highlights the profound complexity of managing refractory PMR—GCA overlap when intolerance to multiple DMARDs prevents steroid withdrawal and exposes patients to cumulative glucocorticoid toxicity.

**Discussion:**

This case illustrates the substantial challenges encountered when managing refractory GCA and PMR in a patient who experienced severe adverse effects to nearly all standard therapies. Treatment required continuous multidisciplinary collaboration and careful balancing of therapeutic benefit against significant toxicity.

Long-term glucocorticoid therapy remains central to inducing remission in GCA, yet recurrent flares often necessitate prolonged tapering. In our patient, repeated relapses led to extended courses lasting over a year, with additional methylprednisolone injections for breakthrough symptoms. The cumulative burden of glucocorticoids resulted in multiple complications, including steroid-induced diabetes, hypertension, obesity, psychological distress, and heightened infection risk. These consequences underscore the importance of effective steroid-sparing strategies, which were unfortunately limited by her intolerance to numerous agents.

Conventional DMARDs—methotrexate, sulphasalazine, leflunomide, hydroxychloroquine, and azathioprine—were trialled sequentially but discontinued due to infections, mucocutaneous toxicity, gastrointestinal complications, or visual deterioration. Biologic therapy with adalimumab was eventually initiated, though other biologics such as abatacept were unavailable. This pattern of intolerance highlights the need for emerging therapies, including liposomal steroids and DAGRs, which may offer anti-inflammatory efficacy with fewer systemic effects. Accurate attribution of adverse effects is essential to avoid prematurely excluding viable treatments.

Tocilizumab, an IL-6 receptor antagonist, has demonstrated superior remission maintenance rates compared to glucocorticoids alone. However, its association with bowel perforation—particularly in patients with diverticular disease—precluded its use here. In such complex cases, shared decision-making is critical, ensuring patients understand risks, benefits, and uncertainties.

Finally, regional MDT involvement was pivotal. Collaboration between rheumatology, gastroenterology, ophthalmology, and vasculitis specialists ensured comprehensive evaluation of therapeutic options and supported nuanced decision-making. This coordinated approach is essential for optimising outcomes in patients with highly refractory PMR—GCA overlap.

**Key learning points:**

Effective management of GCA and PMR requires balancing disease control with treatment tolerability. This case highlights several important learning points relevant to clinicians managing complex vasculitis.

1. Long-term steroid exposure carries significant risk. Prolonged glucocorticoid tapering is often unavoidable due to the relapsing nature of GCA. Courses may extend for years, increasing the likelihood of complications such as cardiovascular disease, diabetes, osteoporosis, infection, and psychological distress. Recognising these risks early reinforces the need for effective steroid-sparing strategies and close monitoring.

2. DMARD intolerance can severely limit remission maintenance. Synthetic and biologic DMARDs are central to maintaining remission, yet intolerance can significantly restrict therapeutic options. In this case, methotrexate caused mucositis and dyspnoea, sulphasalazine caused rash, and several agents triggered diverticulitis. Clinicians must remain vigilant in identifying true drug-related adverse effects to avoid prematurely discontinuing potentially beneficial therapies.

3. Risk–reward discussions are essential in refractory disease. When treatment options become limited by toxicity, shared decision-making becomes critical. Patients must be fully informed of the risks, benefits, and uncertainties surrounding each therapy. Specialist MDT involvement ensures that decisions are supported by multidisciplinary expertise and that all feasible options are considered.

4. Tocilizumab and future IL-6 inhibitors offer promise but require caution. Tocilizumab has strong evidence for maintaining GCA remission, yet its risk of gastrointestinal perforation—particularly in patients with diverticular disease—may limit use. Future IL-6 inhibitors with improved safety profiles may become vital alternatives for patients unable to tolerate conventional DMARDs.

5. Psychological burden must not be overlooked.

